# GLP-1 receptor agonists or SGLT2-inhibitors? Evaluation of a personalized treatment algorithm for individuals with type 2 diabetes: a registry-based cohort study

**DOI:** 10.1055/a-2798-6496

**Published:** 2026-03-25

**Authors:** Tim Mori, Oliver Kuß, Julia K. Mader, Michael Naudorf, Jochen Seufert, Reinhard W. Holl, Stefanie Lanzinger, Julia M. Grimsmann

**Affiliations:** 1 568077 German Diabetes Center Institute for Biometrics and Epidemiology, Düsseldorf, Germany; 2 551467 German Center for Diabetes Research, Neuherberg, Germany; 3 9170 Centre for Health and Society, Heinrich Heine University Düsseldorf, Düsseldorf, Germany; 4 31475 Department of Internal Medicine, Medical University of Graz, Graz, Austria; 5 Diabetes Center Lindlar, Lindlar, Germany; 6 14879 Department of Medicine II, Medical Center – University of Freiburg, Freiburg, Germany; 7 221547 Ulm University Institute of Epidemiology & Medical Biometry, Ulm, Germany

**Keywords:** GLP-1 Receptor Agonists, SLGT2 inhibitors, Personalized medicine, Treatment algorithm, Dynamic weighted survival modelling

## Abstract

**Aims/hypothesis:**

Guidelines recommend GLP-1 receptor agonists (GLP-1-RA) and SGLT2-inhibitors (SGLT2i) for individuals with type 2 diabetes (T2D) at high risk of atherosclerotic cardiovascular disease (ASCVD). In the context of precision medicine, we evaluated a personalized treatment algorithm to guide the initial decision between these therapies.

**Methods:**

Using data from the observational Diabetes Prospective Follow-up registry (Germany/Austria) we studied individuals with T2D who initiated GLP-1-RA (n=1433) or SGLT2i (n=2547) in a multicenter, real-world setting. Baseline characteristics included age, sex, body mass index (BMI), estimated glomerular filtration rate (eGFR), HbA1c, diabetes duration, and history of ASCVD. Non-fatal ASCVD events (myocardial infarction, angina, revascularization, stroke, transient ischemic attack, and peripheral artery disease) were analyzed using dynamic weighted survival modeling to predict the optimal treatment for each individual.

**Results:**

The algorithm predicted 48% of individuals to have better ASCVD outcomes with GLP-1-RA and 52% with SGLT2i. GLP-1-RA-optimal individuals had on average a higher BMI (37 vs 31 kg/m ^2^ ), lower eGFR (71 vs 93 ml/min per 1.73 m ^2^ ) and less history of ASCVD (9 vs 18%) compared to SGLT2i-optimal individuals. However, an internal model validation showed that the predicted optimal treatment did not statistically significantly prolong the average time to a non-fatal ASCVD event compared to the suboptimal treatment (AFT parameter: 1.13; 95% CI: 0.83-1.56; HR: 0.88; 95% CI: 0.64-1.21).

**Conclusions/interpretation:**

The personalized treatment algorithm for GLP-1-RA and SGLT2i did not result in clear individual ASCVD benefits on either drug, a finding consistent with the clinical equipoise reflected in current T2D treatment guidelines.

## Introduction

GLP-1 receptor agonists (GLP-1-RA) and SGLT2-inhibitors (SGLT2i) are important treatment options for type 2 diabetes (T2D) mellitus 1 . Current guidelines recommend SGLT2i for individuals with established heart failure (HF) or chronic kidney disease (CKD) 1 . By contrast, GLP-1-RA are recommended for lowering body weight due to their higher weight loss efficacy 1 . For those at a high risk of atherosclerotic cardiovascular disease (ASCVD), both drugs are recommended without a clear preference 1 2 . In such a setting of clinical equipoise, a precision medicine approach can potentially guide treatment decisions 3 4 .

A recent systematic review from the ADA/EASD Precision Medicine in Diabetes Initiative (PMDI) 5 evaluated evidence on treatment effect heterogeneity for GLP-1-RA and SGLT2i to inform personalized treatment. Regarding cardiovascular outcomes, they found no consistent evidence that clinical features such as age, sex or BMI modify the treatment effect of GLP-1-RA or SGLT2i 5 . The authors argue that this lack of consistency may be due to methodological limitations of the included studies 5 . To address these issues, an “individualized prediction” 6 approach has been proposed, which combines multiple clinical features in a model to predict a person’s drug response 7 . Such a model has recently been developed for GLP-1-RA and SGLT2i to optimize individual 12-month HbA1c response 8 . However, personalized treatment algorithms to optimize ASCVD outcomes are currently lacking.

To address this gap, we evaluated a personalized treatment algorithm for GLP-1-RA and SGLT2i to optimize ASCVD outcomes in individuals with T2D. In this cohort study, we analyzed data of new-users of GLP-1-RA and SGLT2i from diabetes clinics across Germany and Austria. Using a novel statistical method, dynamic weighted survival modelling (DWSurv) 9 , our objectives were to (1) assess whether previously proposed routine clinical features (e. g. age, sex, BMI) modify the cardioprotective effect of GLP-1-RA compared to SGLT2i (discovery), (2) develop an easy-to-use personalized treatment algorithm (prediction) and (3) internally validate the algorithm (confirmation).

## Materials and methods

### Study design and participants

The Prospective Diabetes Follow-up Registry (DPV) is a multicentric database covering 517 diabetes clinics and centers across Germany and Austria 10 . Clinicians use the DPV software to record data on clinical characteristics, treatments, and complications of individuals in routine clinical care. 10 11 12 13 . The DPV initiative was approved by the Ethics Committee of the University of Ulm and data collection was approved by local review boards.

We retrieved data on new-users 14 of either GLP-1-RA or SGLT2i who initiated the treatment between January 2013 and September 2023. Additional inclusion criteria were a diagnosis of T2D, age≥18, and previous metformin treatment. Exclusion criteria included initiating both GLP-1-RA and SGLT2i simultaneously, baseline kidney failure (eGFR<15 mL/min, dialysis, or kidney transplantation), or a recent cardiovascular event within 90 days of treatment initiation. A flow chart of the study cohort is available in the online supplement (sFig. 1). To ensure transparency and reduce biases in this real-world data analysis, we specified a target trial protocol and an emulation strategy 15 , available in the online supplement (sTable 1).

### Outcome

The outcome was time to a non-fatal ASCVD event, defined as a composite of time to myocardial infarction, unstable angina, stable angina, coronary revascularization, stroke, transient ischemic attack or peripheral artery disease. We analyzed non-fatal events, because mortality data was not sufficiently available in the DPV registry. Although stable angina and peripheral artery disease are chronic ASCVD diagnoses rather than acute events, we included them in the composite outcome to capture a broader spectrum of ASCVD complications. Individuals were followed from initiation of GLP-1-RA or SGLT2i treatment until occurrence of a non-fatal ASCVD event, change in treatment regimen, loss to follow-up, or end of the observation period, whichever occurred first.

### Statistical Analysis

#### Dynamic Weighted survival modelling (DWSurv)

DWSurv is a statistical method for developing personalized treatment algorithms in settings with time-to-event outcomes based on real-world data 9 16 . The statistical model underlying DWSurv is a weighted semi-parametric Accelerated Failure Time (AFT) model with interaction terms between clinical features (e. g. eGFR, BMI) and treatment 9 . The resulting algorithm takes an individual’s clinical features as input and outputs a recommended treatment (e. g. GLP-1-RA) to optimize ASCVD outcomes. The personalized treatment algorithm is based on a linear model which makes it similar to well-established prediction models like the Framingham risk score 17 18 .

#### Tailoring variables and confounders

Based on previous literature 5 8 , we assessed whether the following a priori selected clinical features (“tailoring variables”) were associated with a differential treatment response to GLP-1-RA versus SGLT2i: age, sex, BMI, HbA1c, diabetes duration, eGFR, number of current-glucose lowering drugs and history of ASCVD. History of ASCVD was defined analogously to the outcome (non-fatal ASCVD events), but indicated whether an individual had experienced an event prior to treatment initiation. The continuous tailoring variables (age, BMI, HbA1c, diabetes duration, eGFR, number of current glucose-lowering medications) were centered 18 and age and eGFR were scaled to units of 10 years and 15 ml/min per 1.73 m ^2^ , respectively.

The model also included the following baseline characteristics as confounders: systolic blood pressure, diastolic blood pressure, cholesterol, triglycerides, smoking status, lipid lowering treatment, blood pressure lowering treatment, insulin treatment, year of treatment initiation and number of ever-prescribed glucose-lowering drugs. All baseline clinical features were retrieved at the closest available time point prior to treatment initiation, but no more than 12 months prior (18 months for cholesterol due to less frequent measurements). A complete-case analysis was performed, in which only participants with no missing values were included in the analysis. A detailed overview of the DWSurv model specification is available in the online supplement (sTable 2). Documented history of heart failure was very rare in our cohort (only 1% of individuals), possibly due to underreporting in the DPV registry 19 . As a result, it was not possible to include it as a confounder during model development. However, for individuals with a recorded history, heart failure was adjusted for in the internal model validation.

#### Model development

The DPV registry cohort was split into a 60% model development sample (n=2388) and a 40% internal validation sample (n=1592) 7 8 . Before developing the personalized treatment algorithm, we first estimated the treatment effect of GLP-1-RA versus SGLT2i on time to a non-fatal ASCVD event. To do so, we fitted an exponential AFT model that adjusted for all baseline features as confounders but did not include treatment interactions. The treatment effect was interpreted as a multiplicative change, reflecting an acceleration or deceleration of time to a non-fatal ASCVD event under GLP-1-RA compared to SGLT2i.

For the personalized treatment algorithm, we considered two versions of the DWSurv model: 1) a full model, and 2) a parsimonious model. The full model included interaction terms between treatment and all a priori selected tailoring variables. The parsimonious model retained only the three tailoring variables with the largest standardized treatment interactions from the full model. This selection aimed to balance clinical relevance and model parsimony to yield a simple, easy-to-use treatment algorithm. Standard errors and 95% confidence intervals for the DWSurv model coefficients were derived based on 500 bootstrap samples 20 . To predict individualized treatment effects on an absolute time scale, we used the DWSurv model coefficients to predict the time to a non-fatal ASCVD event under either treatment (GLP-1-RA and SGLT2i) and calculated the difference between the two.

#### Internal model validation

Model validation was performed using the concordant-discordant approach of Dennis et al. 7 . This approach evaluates the treatment algorithm by comparing the outcomes of individuals who received their recommended treatment (concordant) to those who did not receive it (discordant). Based on the contrast between recommended and actual treatment, there were four subgroups:

1. GLP-1-RA-optimal individuals who initiated GLP-1-RA treatment (concordant)
2. GLP-1-RA-optimal individuals who initiated SGLT2i treatment (discordant)
3. SGLT2i-optimal individuals who initiated SGLT2i treatment (concordant)
4. SGLT2i-optimal individuals who initiated GLP-1-RA treatment (discordant)

For the model validation, we fit a separate exponential AFT model comparing ASCVD outcomes between concordant treatment (group 1 and 3 combined) and discordant treatment (group 2 and 4 combined). Additionally, we used contrasts to separately compare treatments among GLP-1-RA-optimal individuals (group 1 vs. group 2) and SGLT2i-optimal individuals (group 3 vs. group 4). Validation models were adjusted for all covariates, including tailoring variables and confounders.

#### Software

Data preparation was conducted using SAS (version 9.4) and statistical analyses were performed in R (version 4.3.1) 21 . The DWSurv model was implemented using the DTRreg package (version 1.7) 22 23 . Study reporting follows STROBE guidelines for observational studies 24 as well as BePRECISE guidelines for precision medicine research 25 .

## Results

We identified 3980 eligible individuals with T2D in the DPV registry who initiated GLP-1-RA (n=1433) or SGLT2i (n=2547) ( Table 1 ). Compared to SGLT2i users, GLP-1-RA users were younger (58.3 vs. 63.1 years), more often female (47.8% vs. 37.8%) and had a higher BMI (36.4 vs. 32.2 kg/m ^2^ ). The median (Q1; Q3) follow-up time after treatment initiation was 9.2 (3; 17.4) months. During follow-up, 437 (11%) individuals experienced a non-fatal ASCVD event (GLP-1-RA: 121 [8%] events, SGLT2i: 316 [12%] events).

**Table:** **Table 1** Baseline clinical characteristics of individuals initiating GLP-1 receptor agonists (GLP-1-RA) and SGLT2-inhibitors (SGLT2i) treatment in the Diabetes Prospective Follow-up (DPV) registry.

	GLP-1-RA new users (n=1433)	SGLT2i new users (n=2547)	Difference (95% CI)
Age, years	58.3 [11.7]	63.1 [11.4]	4.8 (4.0; 5.5)
Sex			
Male	748 (52.2%)	1584 (62.2%)	10.0% (6.7; 13.2)
Female	685 (47.8%)	963 (37.8%)	
BMI, kg/m ^2^	36.4 [6.9]	32.2 [6.1]	–4.2 (–4.6; –3.8)
HbA1c,%	7.95 [1.57]	7.64 [1.37]	–0.31 (–0.41; –0.21)
Diabetes duration, years	10.8 [8.1]	11.6 [8.5]	0.7 (0.2; 1.3)
eGFR, ml/min per 1.73m ^2^	85.1 [21.3]	81.3 [19.9]	–3.8 (–5.2; –2.5)
Year of treatment initiation, 0=2013, … 10=2023	4.34 [3.09]	4.78 [2.66]	0.45 (0.26; 0.64)
Number of current glucose-lowering drugs	1.69 [0.88]	1.82 [0.92]	0.13 (0.07; 0.19)
Number of ever prescribed glucose-lowering drugs	2.11 [1.00]	2.29 [1.11]	0.19 (0.12; 0.25)
History of ASCVD*			
Previous event	175 (12.2%)	397 (15.6%)	3.4% (1.1; 5.6)
No previous event	1258 (87.8%)	2150 (84.4%)	
History of Heart Failure			
Yes	5 (0.3%)	35 (1.4%)	1.1% (0.5; 1.7)
No	1428 (99.7%)	2512 (98.6%)	
Systolic blood pressure, mmHg	137.8 [16.3]	136.7 [15.6]	–1.1 (–2.1; –0.1)
Diastolic blood pressure, mmHg	82.3 [9.9]	80.4 [9.5]	–1.9 (–2.5; –1.2)
Cholesterol, mg/dl	189.0 [44.4]	184.6 [44.9]	–4.4 (–7.3; –1.5)
Triglycerides, mg/dl	217.8 [120.8]	209.0 [126.0]	–8.8 (–16.8; –0.9)
Smoking status			
Active smoker	86 (6.0%)	148 (5.8%)	–0.2% (–1.8; 1.4)
Not an active smoker	1347 (94.0%)	2399 (94.2%)	
Lipid lowering drug prescription			
Yes	378 (26.4%)	872 (34.2%)	7.9% (5.9; 10.8)
No	1055 (73.6%)	1675 (65.8%)	
Blood pressure lowering drug prescription			
Yes	675 (47.1%)	1342 (52.7%)	5.6% (2.3; 8.9)
No	758 (52.9%)	1205 (47.3%)	
Insulin treatment			
Yes	661 (46%)	1100 (43%)	–2.9% (–6.2; 0.3)
No	771 (54%)	1447 (57%)	

Data are mean [SD] and number (%). *Myocardial infarction, unstable angina, stable angina, coronary revascularization, stroke, transient ischemic attack or peripheral artery disease. ASCVD: atherosclerotic cardiovascular disease; BMI: body mass index; DPV: Diabetes Prospective Follow-up; eGFR: estimated Glomerular Filtration Rate; GLP-1-RA: GLP-1 receptor agonists; HbA1c: hemoglobin A1c; SGLT2i: SGLT2-inhibitors.

### Model development

The 60% model development sample (n=2389) included 843 new users of GLP-1-RA and 1545 new users of SGLT2i (sTable 3). Based on the exponential AFT model without interactions, initiation of GLP-1-RA slightly decelerated time to a non-fatal ASCVD event by a factor of 1.24 (95% CI: 0.92–1.69) compared to SGLT2i (HR: 0.80, 95% CI: 0.59–1.09). Similarly, the intercept of the DWSurv model indicated that GLP-1-RA treatment decelerated the time to a non-fatal ASCVD event by a factor of 1.30 (95% CI: 0.66–2.58) for an average person in the DPV registry.

In the DWSurv model, however, the predicted treatment effect varies by individual characteristics, as the model includes interaction terms between treatment and the tailoring variables ( Table 2 ). For example, history of ASCVD at treatment initiation reduced the treatment effect of GLP-1-RA compared to SGLT2i by a factor of 0.39 (95% CI: 0.13–1.13). For such a person, SGLT2i would be favored, as time to a new non-fatal ASCVD event would be accelerated by a factor of 1.30×0.39=0.51 under GLP-1-RA treatment. Similarly, a 15 ml/min per 1.73 m ^2^ higher eGFR reduced the treatment effect of GLP-1-RA by a factor of 0.58 (95% CI: 0.40–0.84). In contrast, a 1 kg/m ^2^ higher BMI increased the treatment effect of GLP-1-RA by a factor of 1.05 (95% CI: 0.97–1.14). Age, sex, and diabetes duration did not seem to modify the treatment effect of GLP-1-RA compared to SGLT2i.

**Table:** **Table 2** Estimated treatment interactions (incl. 95% CI) of the dynamic weighted survival (DWSurv) model in terms of multiplicatively changing (accelerating or decelerating) time to a non-fatal atherosclerotic cardiovascular disease (ASCVD) event under GLP-1 receptor agonists (GLP-1-RA) treatment. Coefficients>1 favor GLP-1-RA treatment and coefficients<1 favor SGLT2-inhibitors (SGLT2i) treatment. Estimates are shown for the full model and the parsimonious model.

	Full model		Parsimonious model	
Tailoring variable	Coefficient	95% CI	Coefficient	95% CI
Intercept*	1.30	(0.66; 2.58)	1.29	(0.81; 2.03)
History of ASCVD	0.39	(0.13; 1.13)	0.43	(0.16; 1.14)
Age, 10 years	0.90	(0.56; 1.47)	-	-
Sex, male	0.99	(0.44; 2.25)	-	-
eGFR, 15 ml/min per 1.73 m ^2^	0.58	(0.40; 0.84)	0.60	(0.46; 0.79)
BMI, kg/m ^2^	1.05	(0.97; 1.14)	1.05	(0.98; 1.12)
HbA1c,%	0.87	(0.61; 1.25)	-	-
Diabetes duration, years	1.01	(0.96; 1.05)	-	-
Number of current glucose lowering drugs	1.18	(0.73; 1.90)	-	-

*The intercept corresponds to the estimated treatment effect for a reference person (no history of ASCVD, female) with an average clinical profile as observed in the DPV cohort (Age 61 years, eGFR 83 ml/min per 1.73 m ^2^ , BMI 33.6 kg/m ^2^ , HbA1c 7.7%, diabetes duration 11 years, 2 glucose-lowering drugs currently prescribed). ASCVD: atherosclerotic cardiovascular disease; BMI: body mass index; DWSurv: dynamic weighted survival; eGFR: estimated Glomerular Filtration Rate; GLP-1-RA: GLP-1 receptor agonists; HbA1c: hemoglobin A1c; SGLT2i: SGLT2-inhibitors.

**Fig. 1:**
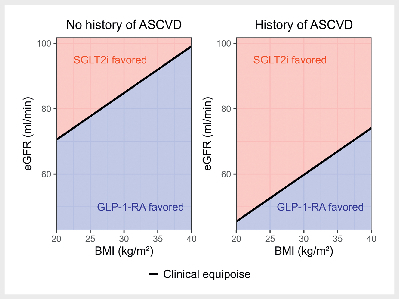
Personalized treatment algorithm for selecting between GLP-1 receptor agonists (GLP-1-RA) and SGLT2-inhibitors (SGLT2i) to prevent non-fatal atherosclerotic cardiovascular disease (ASCVD) events based on the parsimonious dynamic weighted survival (DWSurv) model. The algorithm identifies an optimal linear decision rule incorporating body mass index (BMI), estimated Glomerular Filtration Rate (eGFR) and history of ASCVD. Note that for some individuals (e. g. a person with a history of ASCVD, BMI 30 kg/m ^2^ , eGFR 60 ml/min per 1.73m ^2^ ) both treatments are predicted to perform similarly well. However, the further an individual’s clinical profile is away from this “clinical equipoise”, the higher their predicted benefit on the recommended treatment. The exact formula for the personalized treatment algorithm is available in the online supplement (sFig. 2). ASCVD: atherosclerotic cardiovascular disease; BMI: body mass index; DWSurv: dynamic weighted survival; eGFR: estimated Glomerular Filtration Rate; GLP-1-RA: GLP-1 receptor agonists; SGLT2i: SGLT2-inhibitors.

**Fig. 2:**
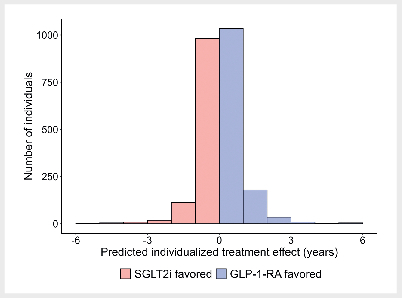
Predicted individualized treatment effects (predicted difference in time to a non-fatal atherosclerotic cardiovascular disease (ASCVD) event) for individuals in the model development sample based on the parsimonious dynamic weighted survival (DWSurv) model. Positive values reflect a predicted treatment benefit on GLP-1 receptor agonists (GLP-1-RA) treatment and negative values reflect a predicted treatment benefit on SGLT2-inhibitors (SGLT2i) treatment. ASCVD: atherosclerotic cardiovascular disease; DWSurv: dynamic weighted survival; GLP-1-RA: GLP-1 receptor agonists; SGLT2i: SGLT2-inhibitors.

Based on the standardized coefficients of the full model (sTable 4), the parsimonious DWSurv model included only history of ASCVD, eGFR and BMI as tailoring variables. The resulting personalized treatment algorithm ( Fig. 1 ) recommends GLP-1-RA for individuals with a higher BMI, lower eGFR and no history of ASCVD. Conversely, it recommends SGLT2i for individuals with a lower BMI, higher eGFR and a history of ASCVD. While only the treatment interaction with eGFR was statistically significant (see Table 2 ), the interactions with history of ASCVD and BMI might still be clinically relevant despite statistical uncertainty. Moreover, the combination of several predictors in the treatment algorithm might yield relevant benefits, even if individual predictors did not show a statistically significant treatment interaction. The exact formula for the personalized treatment algorithm is available in the online supplement (sFig. 2). Across the 2389 individuals in the model development sample, the median (Q1; Q3) predicted individualized treatment effect on the absolute time scale was 0.4 (–3.8; 4.9) months ( Fig. 2 ). In other words, clinical equipoise or only small benefits were predicted for most individuals in terms of time to a non-fatal ASCVD event.

### Internal model validation

The 40% internal model validation sample (n=1592) included 590 new users of GLP-1-RA and 1002 new users of SGLT2i (sTable 5). Based on the parsimonious treatment algorithm, 851 individuals (53%) were recommended GLP-1-RA treatment and 741 (47%) were recommended SGLT2i treatment. GLP-1-RA-optimal individuals had on average a higher BMI (35.7 vs 32 kg/m ^2^ ), lower eGFR (70 vs 97 ml/min per 1.73 m ^2^ ) and fewer previous ASCVD events (9% vs 22%) compared to SGLT2i-optimal individuals (sTable 6). In terms of actual treatment received, 474 (64%) SGLT2i-optimal individuals received concordant treatment, compared to only 323 (38%) GLP-1-RA-optimal individuals.

**Fig. 3:**
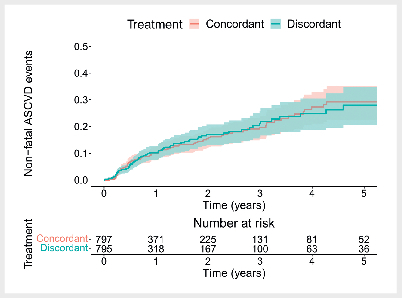
Kaplan-Meier curves comparing the occurrence of non-fatal atherosclerotic cardiovascular disease (ASCVD) events between concordant individuals (recommended treatment initiated) and discordant individuals (non-recommended treatment initiated) in the 40% internal model validation sample. ASCVD: atherosclerotic cardiovascular disease.

In the concordant-discordant analysis ( Fig. 3 ), concordant (i. e. optimal) treatment did not statistically significantly prolong the average time to a non-fatal ASCVD event compared to discordant (i. e. suboptimal) treatment (AFT parameter: 1.13; 95% CI: 0.83–1.56; HR: 0.88; 95% CI: 0.64–1.21). The results were similar when assessing the effects of concordant treatment separately for GLP-1-RA-optimal individuals and SGLT2i-optimal individuals. For GLP-1-RA-optimal individuals, GLP-1-RA treatment did not statistically significantly prolong the average time to a non-fatal ASCVD event compared to SGLT2i treatment (AFT parameter: 1.23; 95% CI: 0.76–1.98; HR: 0.81; 95% CI: 0.51–1.31). Similarly, for SGLT2i-optimal individuals, SGLT2i treatment did not statistically significantly prolong the average time to a non-fatal ASCVD event compared to GLP-1-RA treatment (AFT parameter: 1.13; 95% CI: 0.68–1.88; HR: 0.88; 95% CI: 0.53–1.46). Although the direction of the estimated treatments effects favored the recommended treatment, the wide confidence intervals indicate substantial statistical uncertainty. Therefore, we cannot conclude superiority of concordant compared to discordant treatment. The internal model validation results for the full model were similar and are available in the online supplement (sTable 7).

## Discussion and conclusions

In this study, we developed a personalized treatment algorithm for GLP-1-RA and SGLT2i to optimize ASCVD outcomes in individuals with T2D. The algorithm favored GLP-1-RA for those with a higher BMI, and SGLT2i for those with a history of ASCVD and higher eGFR. However, in the internal model validation, treatment aligned with these recommendations did not result in clearly improved ASCVD outcomes.

It is surprising that GLP-1-RA might be preferred over SGLT2i for ASCVD prevention in individuals with lower kidney function, considering the established role of SGLT2i in treating chronic kidney disease 1 . Interestingly, however, this aligns with the HbA1c-based personalized treatment algorithm of Cardoso et al. 8 , which also favors GLP-1-RA over SGLT2i in individuals with lower kidney function. Regarding HbA1c response, the lower efficacy of SGLT2i at lower eGFR has been reported in several studies 5 . However, for cardiovascular outcomes, the PMDI systematic review 5 as well as a recent network meta-analysis 26 report no consistent evidence of differential effectiveness by baseline kidney function. Given this inconsistent evidence and the low proportion of individuals with eGFR<60 ml/min per 1.73m ^2^ in our study cohort (~ 15%), our findings should be treated with caution.

The finding that SGLT2i might be favored over GLP-1-RA for individuals with a history of ASCVD is supported by a large-scale, propensity-score matched analysis of non-fatal ASCVD events in U.S. claims data 27 . However, the PMDI systematic review 5 finds that overall the evidence for treatment effect modification by history of CVD is inconsistent for both GLP-1-RA and SGLT2i. Regarding BMI, however, they report some evidence that GLP-1-RA have higher cardiovascular efficacy for individuals with higher BMI at treatment initiation 5 . This is plausible given their strong weight-lowering efficacy 1 and approval as anti-obesity medications 28 . Another potential effect modifier for GLP-1-RA is ethnicity, with larger cardiovascular benefits having been observed in Asian populations 5 . Unfortunately, data on ethnicity was not sufficiently available in the German / Austrian DPV registry, so we were unable to investigate it in the current study.

All in all, the PMDI systematic review 5 suggests that so far there is little consistent evidence that individual clinical features modify the cardioprotective effects of GLP-1-RA and SGLT2i. Therefore, it is perhaps not surprising that our internal model validation did not show clear benefits for individuals treated according to our personalized treatment algorithm. Similarly, the personalized treatment algorithm of Cardoso et al. 8 , while improving glycemic outcomes, did not show improvements in cardiovascular outcomes. Instead, they found clinical equipoise between GLP-1-RA and SGLT2i regarding new-onset MACE, regardless of the predicted individual glycemic benefit of the respective drug 8 . This equipoise is further supported by a recent large-scale comparative effectiveness study of 244,694 SGLT2i initiators and 123,991 GLP-1-RA initiators, which found no differences in 3-point or 4-point MACE between the two drugs 29 .

A major strength of our study is that we addressed several “evidence gaps” identified by the PMDI systematic review on treatment effect heterogeneity of GLP-1-RA and SGLT2i 5 . First, we analyzed real-world data from the DPV registry, allowing for a head-to-head comparative effectiveness evaluation of GLP-1-RA and SGLT2i in routine clinical practice 5 . Second, treatment effect modifiers were selected a priori 5 8 and evaluated jointly in a model to predict individual treatment response. Third, instead of analyzing glycemic outcomes, we focused on the prevention of cardiovascular outcomes, for which GLP-1-RA and SGLT2i are major components of current guidelines 1 5 . Finally, using the novel DWSurv model 9 , we developed a personalized treatment algorithm that is easy to understand and offers clear clinical interpretation, which enhances its usefulness as a decision support tool 4 30 .

Given the real-world nature of our study, several limitations should be acknowledged. The major limitation is that only non-fatal ASCVD events could be analyzed, as mortality data were not sufficiently available in the DPV registry. Second, the relatively short median follow-up time of 9.2 months limits conclusions about long-term ASCVD risk reduction. Third, while key CVD risk factors like blood pressure and cholesterol were included, data on lifestyle factors such as physical activity, diet, and alcohol consumption were not exactly documented in the DPV registry and therefore not studied. Additionally, history of heart failure was not well-documented 19 , and since SGLT2i play a significant role in its treatment, confounding by indication may have influenced model development. Fourth, the time period that was analyzed (January 2013 until September 2023) included early years, when the most powerful GLP-1-RA were not available. Finally, while we validated our model internally, we did not carry out an external model validation. However, since the validation showed no clear ASCVD benefits within the original population in which the model was developed, it is unlikely that they would emerge in a different population. In terms of generalizability, the DPV registry can be regarded as representative for adult individuals with T2D treated in routine diabetes specialist care in Germany and Austria. Note that benchmarking against current practice 25 was not possible, as there are currently no individualized prediction models for GLP-1-RA and SGLT2i to optimize ASCVD outcomes.

As of now, it is unclear whether the lack of consistent findings for individualized ASCVD prevention using GLP-1-RA and SGLT2i is due to methodological limitations of existing studies 4 5 25 or if the potential of such precision medicine approaches is smaller than previously anticipated 31 32 33 . Future research should focus on the priorities outlined in the PMDI consensus report on gaps and opportunities for precision diabetes medicine 4 and follow the BePRECISE reporting guidelines 25 to improve the current evidence base. In conclusion, our personalized treatment algorithm for GLP-1-RA and SGLT2i did not result in clear individual ASCVD benefits of either drug, a finding consistent with the clinical equipoise reflected in current T2D treatment guidelines 1 .

## Data availability

Due to protection of patient privacy and the specifications in the patient/parent consent form, it is not allowed to share patient level data with researchers outside Ulm University. However, aggregated data are available and collaboration based on remote data access is also possible.

## Code availability

The analysis code of this study is available upon reasonable request.

## Ethical approval and human rights

The DPV Initiative as well as the analyses of anonymized data have been approved by the ethics committee at the University of Ulm (314/21). Participating centers obtained local data protection approval. This study has therefore been performed in accordance with the ethical standards laid down in an appropriate version of the 1964 Declaration of Helsinki.

## Informed consent

All patients being enrolled into the DPV registry provided informed consent.

TM is supported by the EASD mentorship program. The German Diabetes Center is funded by the German Federal Ministry of Health and the Ministry of Culture and Science of the state of North Rhine-Westphalia. The German Center for Diabetes Research is funded by the German Federal Ministry of Education and Research. Financial support for DPV was provided by the German Center for Diabetes Research (DZD, grant number 82DZD14H03). Additional funding was provided by the REDDIE project (grant agreement 101095556). The sole responsibility for the content of this publication lies with the authors. The funding sources had no role in study design, data collection, data analysis, data interpretation, or writing of the report.

## Supplementary Material

Supplementary Material Zusatzmaterial
