# Supplementary material for: GLP-1 receptor agonists or SGLT2-inhibitors? Evaluation of a personalized treatment algorithm for individuals with type 2 diabetes: a registry-based cohort study
Source: Exp Clin Endocrinol Diabetes. 2026 Mar 25;134(3):79–87. doi: 10.1055/a-2798-6496 (PMC13016828; doi:10.1055/a-2798-6496)
Supplement: Supplementary file 1 — Supplementary Material [file 10-1055-a-2798-6496-12-2025-0341-dia.pdf]

GLP-1 receptor agonists or SGLT2-inhibitors? Evaluation of a personalized treatment algorithm for individuals with type 2 diabetes: a registry-based cohort study

T. Mori[1,2], O. Kuss[1,2,3], J.K. Mader[4], M. Naudorf[5], J. Seufert[6], R.W. Holl[2,7], S. Lanzinger[2,7], J.M. Grimsmann[2,7], DPV initiative

[1] Institute for Biometrics and Epidemiology, German Diabetes Center, Leibniz Center for Diabetes Research at Heinrich Heine University Düsseldorf, Düsseldorf, Germany

[2] German Center for Diabetes Research (DZD), München-Neuherberg, Germany

[3] Centre for Health and Society, Medical Faculty and University Hospital Düsseldorf, Heinrich Heine University Düsseldorf, Germany

[4] Division of Endocrinology and Diabetology, Department of Internal Medicine, Medical University of Graz, Graz, Austria

[5] Diabetes Center Lindlar, Lindlar, Germany

[6] Division of Endocrinology and Diabetology, Department of Medicine II, Medical Center - University of Freiburg, Faculty of Medicine, University of Freiburg, Freiburg, Germany

[7] Institute of Epidemiology and Medical Biometry, Ulm University, Ulm, Germany

Corresponding author:

Tim Mori (tim.mori@ddz.de)

Supplement – Methods

sFig. 1 – Flow chart showing the data extraction from the Diabetes Prospective Follow-up (DPV) registry to the study cohort. BMI: body mass index; DPV: Diabetes Prospective Follow-up; GLP-1 RA: GLP-1 receptor agonists; HbA1c: hemoglobin A1c; HDL: High-Density Lipoprotein; LDL: Low-Density Lipoprotein; SGLT2i: SGLT2-inhibitors.

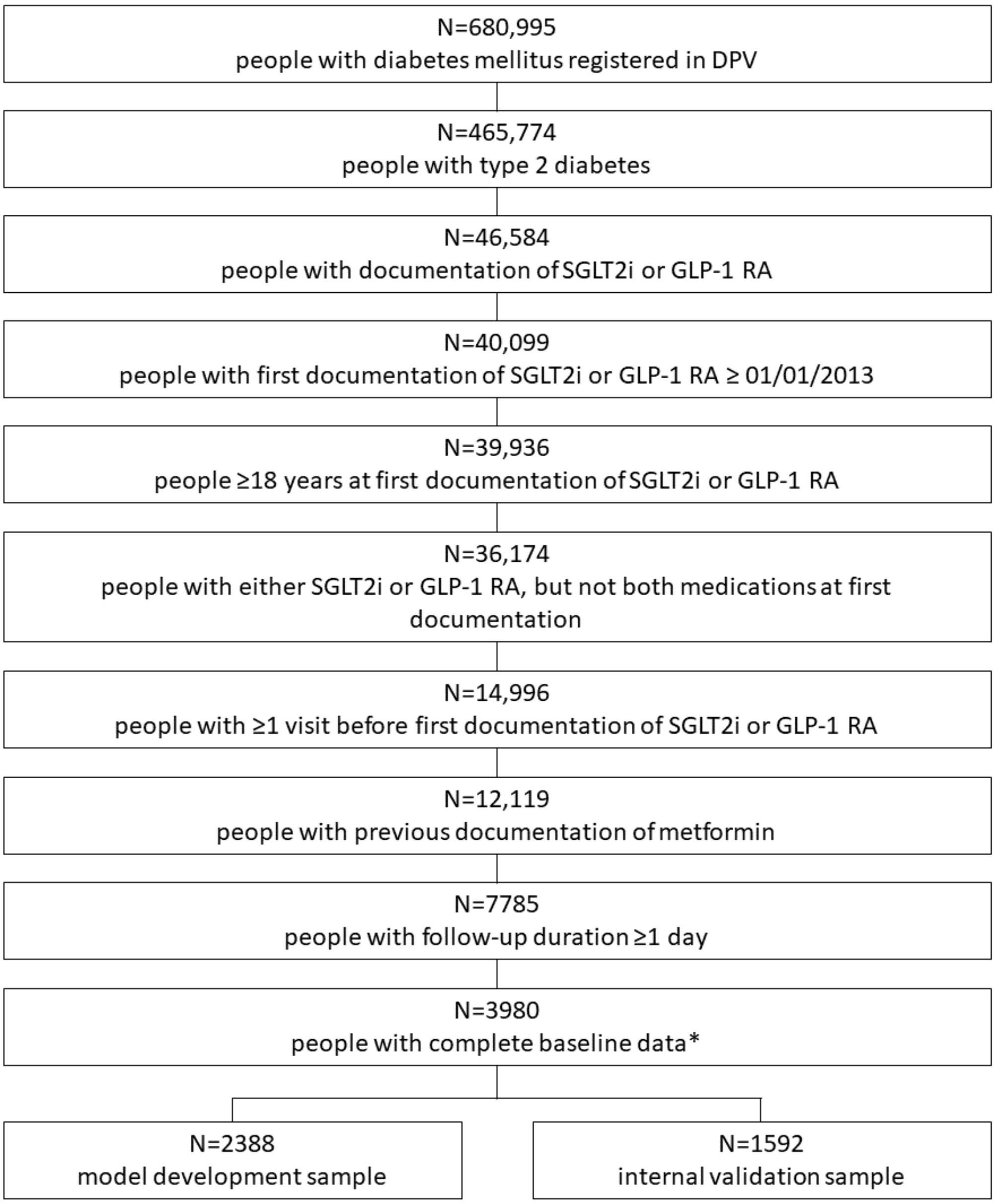

*HbA1c, BMI, systolic and diastolic blood pressure, antihypertensive medication, lipid-lowering medication, smoking (documented at least once in past 12 months); triglycerides, HDL, LDL and total cholesterol (documented at least once in past 18 months before first documentation of SGLT2i or GLP-1 RA)

sTable 1 – Target trial emulation protocol. The blue text highlights where the emulated trial based on the Diabetes Prospective Follow-up (DPV) registry data differs from the target trial.

	**Target Trial**	**Emulated Trial**
**Aim of the study**	(1) Assess to what extent routine clinical features (e.g. age, sex, BMI) modify the cardioprotective treatment effect of GLP-1-RA compared to SGLT2i<br>(2) Develop an easy-to-use personalized treatment algorithm<br>(3) Internally validate the algorithm	Same.
**Eligibility criteria**	Inclusion:<br>1) T2DM diagnosis<br>2) > 18 years old<br>3) Previous treatment with Metformin<br><br>Exclusion:<br>1) End-stage renal disease (defined by eGFR < 15)<br>2) Recent cardiovascular event 90 days prior to treatment initiation<br>3) Previous treatment with either GLP-1 RA or SGLT2i	Inclusion:<br>Same.<br><br>4) Individuals must be included in the DPV registry. I.e. be treated in a specialist clinic or diabetologist practice, which uses the DPV software.<br>5) Real-world prescription of either SGLT2i or GLP-1 RA by diabetologist.<br><br>Exclusion:<br>Same.<br><br>4) Simultaneous initiation of both SGLT2i and GLP-1 RA
**Treatment strategies**	A) Sustained GLP-1 RA treatment<br>B) Sustained SGLT2i treatment<br><br>Changes to treatment regime: Addition of other drugs (*not* GLP-1 RA or SGLT2i) is allowed. Discontinuation of Metformin and other drugs that were previously used (not GLP-1 RA or SGLT2i) is allowed.	Same.<br><br>Treatment is considered as “sustained” if prescriptions were still present at follow-up visits of a given individual. Brief discontinuations (-up to 6 months) were allowed, if prescriptions were present again at subsequent visits. This was done, because a single visit without a prescription is likely to be a recording error in the medication records.
**Treatment assignment procedures**	At baseline, participants will be randomly assigned to either drug (A) or (B).	Randomization is emulated via adjustment for measured baseline confounders by means of weighting (overlap weights). For this purpose, a treatment model (logistic regression) is fitted to derive treatment propensity scores.
**Follow-up period**	Follow-up starts at randomization and ends at diagnosis of ASCVD event, **death**, loss to follow-up, 5	Follow-up starts at randomization and ends at diagnosis of non-fatal ASCVD event, loss to follow-up, 5

	years after randomization, whichever occurs first.	years after randomization, whichever occurs first.
**Outcome**	ASCVD event or death within 5 years of randomization.	Non-fatal ASCVD event within 5 years of randomization.<br><br>Mortality is not well documented in the DPV registry. Therefore, it could not be analyzed and the outcome (non-fatal ASCVD events) differs from the outcome in the target trial.
**Causal contrast of interest**	Per-protocol-effect. Effect of receiving treatment (A) according to protocol compared to receiving treatment (B).<br><br>Individuals who did not sustain treatment (e.g. discontinuation, add-on of comparator drugs) are artificially censored. Censoring weights are used to reflect the contributions of censored individuals via uncensored individuals with similar covariate patterns.	Same.
**Statistical analysis plan**	Dynamic weighted survival modelling (DWSurv) to estimate an optimal personalized treatment algorithm in the presence of right-censoring.	Same.<br><br>In order to adjust for confounding, measured confounders are included in the outcome model as well as in the treatment model to derive overlap weights.

ASCVD: atherosclerotic cardiovascular disease; BMI: body mass index; DPV: Diabetes Prospective Follow-up; DWSurv: dynamic weighted survival; GLP-1 RA: GLP-1 receptor agonists; SGLT2i: SGLT2-inhibitors; T2DM: type 2 diabetes mellitus.

References

Braitmaier, M., & Didelez, V. (2022). Emulierung von „target trials “mit Real-world-Daten. *Prävention und Gesundheitsförderung*, 1-8. https://doi.org/10.1007/s11553-022-00967-9

Hernán, M. A., & Robins, J. M. (2010). Causal Inference: What If. Boca Raton: Chapman & Hall/CRC

Hernán, M. A., & Hernández-Díaz, S. (2012). Beyond the intention-to-treat in comparative effectiveness research. *Clinical trials*, *9*(1), 48-55. https://doi.org/10.1177/1740774511420743

Hernán, M. A., & Robins, J. M. (2016). Using big data to emulate a target trial when a randomized trial is not available. *American journal of epidemiology*, *183*(8), 758-764. https://doi.org/10.1093/aje/kwv254

Simoneau, G., Moodie, E. E., Azoulay, L., & Platt, R. W. (2020). Adaptive treatment strategies with survival outcomes: an application to the treatment of type 2 diabetes using a large observational database. *American Journal of Epidemiology*, 189(5), 461-469. https://doi.org/10.1093/aje/kwz272

Simoneau, G., Moodie, E. E., Nijjar, J. S., Platt, R. W., & Scottish Early Rheumatoid Arthritis Inception Cohort Investigators. (2020). Estimating optimal dynamic treatment regimes with survival outcomes. *Journal of the American Statistical Association*, *115*(531), 1531-1539 https://doi.org/10.1080/01621459.2019.1629939

DWSurv model specification

Tailoring variables:
History of atherosclerotic cardiovascular disease (ASCVD)
Age
Sex
Estimated Glomerular Filtration Rate (eGFR)
Body mass index (BMI)
Hemoglobin A1c (HbA1c)
Diabetes duration
Current number of glucose-lowering medications

Confounders:
Number of ever prescribed glucose-lowering medications
Systolic blood pressure
Diastolic blood pressure
Lipid-lowering drug prescription
Blood pressure-lowering drug prescription
Insulin treatment
Smoking status
Cholesterol
Triglycerides
Year of treatment initiation

sTable 2 – Specification of the dynamic weighted survival (DWSurv) model.

	**Full Model**	**Parsimonious Model**
**Tailoring Variables**	All tailoring variables	History of ASCVD + eGFR + BMI
**Variables in treatment-free model**	All tailoring variables + confounders	Same
**Variables in treatment model**	All tailoring variables + confounders	Same
**Variables in censoring model**	Treatment indicator (GLP-1-RA / SGLT2i) + All tailoring variables + confounders	Same

ASCVD: atherosclerotic cardiovascular disease; BMI: body mass index; DWSurv: dynamic weighted survival; eGFR: estimated Glomerular Filtration Rate; GLP-1-RA: GLP-1 receptor agonists; SGLT2i: SGLT2-inhibitors.

Supplement – Results

sTable 3 Baseline clinical characteristics of individuals initiating GLP-1 receptor agonists (GLP-1-RA) and SGLT2-inhibitors (SGLT2i) treatment in the 60% model development sample from the Diabetes Prospective Follow-up (DPV) registry.

	**GLP-1-RA new users (n=843)**	**SGLT2i new users (n=1545)**
Age, years	58 [11.7]	63 [11.4]
Sex		
Male	439 (52%)	958 (62%)
Female	404 (48%)	587 (38%)
BMI, kg/m^2	36.5 [7.0]	32.0 [6.1]
HbA1c, %	7.9 [1.5]	7.6 [1.4]
Diabetes duration, years	10.7 [8.0]	11.7 [8.6]
eGFR, ml/min per 1.73m^2	85 [21]	81 [20]
Year of treatment initiation, 0 = 2013, … 10 = 2023	4.1 [3.1]	4.8 [2.7]
Number of current glucose-lowering drugs	1.7 [0.9]	1.8 [0.9]
Number of ever prescribed glucose-lowering drugs	2.1 [1.0]	2.3 [1.1]
History of ASCVD*		
Previous event	102 (12%)	235 (15%)
No previous event	741 (88%)	1310 (85%)
History of Heart Failure		
Yes	2 (0.2%)	21 (1.4%)
No	841 (99.8%)	1524 (98.6%)
Systolic blood pressure, mmHg	138 [16]	137 [15]
Diastolic blood pressure, mmHg	82 [10]	81 [10]
Cholesterol, mg/dl	189 [43]	184 [45]
Triglycerides, mg/dl	220 [121]	206 [124]
Smoking status		
Active smoker	49 (6%)	79 (5%)
Not an active smoker	794 (94%)	1466 (95%)
Lipid lowering drug prescription		
Yes	232 (28%)	529 (34%)
No	611 (72%)	1016 (66%)
Blood pressure lowering drug prescription		
Yes	406 (48%)	803 (52%)
No	437 (52%)	742 (48%)
Insulin treatment		
Yes	388 (46%)	671 (43%)
No	505 (54%)	874 (57%)

Data are mean [SD] and number (%)

*Myocardial infarction, unstable angina, stable angina, coronary revascularization, stroke, transient ischemic attack or peripheral artery disease

ASCVD: atherosclerotic cardiovascular disease; BMI: body mass index; DPV: Diabetes Prospective Follow-up; eGFR: estimated Glomerular Filtration Rate; GLP-1-RA: GLP-1 receptor agonists; HbA1c: hemoglobin A1c; SGLT2i: SGLT2-inhibitors.

sTable 4 Standardized coefficients (incl. 95% CI) of the full dynamic weighted survival (DWSurv model). Coefficients > 1 favor GLP-1 receptor agonists (GLP-1-RA) treatment and coefficients < 1 favor SGLT2-inhibitors (SGLT2i) treatment.

	**Full model**	
**Tailoring variable**	Coefficient	95% CI
Intercept*	1.31	(0.89; 1.91)
History of ASCVD	0.39	(0.14; 1.08)
Age, standardized	0.89	(0.57; 1.38)
Sex, male	0.99	(0.50; 1.97)
eGFR, standardized	0.48	(0.32; 0.72)
BMI, standardized	1.40	(1.00; 1.98)
HbA1c, standardized	0.82	(0.57; 1.18)
Diabetes duration, standardized	1.04	(0.80; 1.38)
Number of current glucose lowering drugs, standardized	1.16	(0.82; 1.64)

*The intercept corresponds to the estimated treatment effect for a reference person (no history of ASCVD, female) with an average clinical profile as observed in the DPV cohort (Age 61 years, eGFR 83 ml/min per 1.73m2, BMI 33.6 kg/m2, HbA1c 7.7%, diabetes duration 11 years, 2 glucose-lowering drugs currently prescribed).

ASCVD: atherosclerotic cardiovascular disease; BMI: body mass index; DWSurv: dynamic weighted survival; eGFR: estimated Glomerular Filtration Rate; GLP-1-RA: GLP-1 receptor agonists; HbA1c: hemoglobin A1c; SGLT2i: SGLT2-inhibitors.

Full model:

Treat patient i with **GLP-1-RA**, if

1.30
* 0.39 * **History of ASCVD**$_i$
* 0.90 * age$_i$
* 0.99 * sex$_i$
* 0.58 * **eGFR**$_i$
* 1.05 * **BMI**$_i$
* 0.87 * HbA1c$_i$
* 1.01 * Diabetes duration$_i$
* 1.18 * Number of current glucose-lowering drugs$_i$

> 1

Otherwise, treat with **SGLT2i**.

Parsimonious model:

Treat patient i with **GLP-1-RA**, if

1.29
* 0.43 * **History of ASCVD**$_i$
* 0.60 * **eGFR**$_i$
* 1.05 * **BMI**$_i$

> 1

Otherwise, treat with **SGLT2i**.

sFig. 2 Personalized treatment algorithm based on the full and the parsimonious version of the dynamic weighted survival (DWSurv) model. The numerical output corresponds to the predicted individual treatment effect of GLP-1 receptor agonists (GLP-1-RA) treatment compared to SGLT2-inhibitors (SGLT2i) treatment. If it is larger than 1, GLP-1-RA treatment is predicted to prolong the time to a non-fatal atherosclerotic cardiovascular disease (ASCVD) event. If it is smaller than 1, GLP-1-RA treatment is predicted to shorten the time to a non-fatal ASCVD event. Note that estimated Glomerular Filtration Rate (eGFR) needs to be scaled to 15 ml/min per 1.73 m^2 and age to 10 years. Moreover, the continuous variables need to be centered with respect to the following mean values from the Diabetes Prospective Follow-up (DPV) model development cohort: age = 61.4, BMI = 33.6 kg/m^2, HbA1c = 7.72%, diabetes duration = 11.3, eGFR = 82.5 ml/min per 1.73m^2, number of current glucose-lowering medications = 1.8). ASCVD: atherosclerotic cardiovascular disease; BMI: body mass index; DWSurv: dynamic weighted survival; eGFR: estimated Glomerular Filtration Rate; GLP-1-RA: GLP-1 receptor agonists; HbA1c: hemoglobin A1c; SGLT2i: SGLT2-inhibitors.

sTable 5 Baseline clinical characteristics of individuals initiating GLP-1 receptor agonists (GLP-1-RA) and SGLT2-inhibitors (SGLT2i) treatment in the 40% model validation sample from the Diabetes Prospective Follow-up (DPV) registry.

	**GLP-1-RA new users (n=590)**	**SGLT2i new users (n=1002)**
Age, years	58 [12]	63 [11]
Sex		
Male	309 (52%)	626 (62%)
Female	281 (48%)	376 (38%)
BMI, kg/m^2	36.6 [6.8]	32.5 [6.2]
HbA1c, %	8.0 [1.6]	7.7 [1.4]
Diabetes duration, years	11.0 [8.2]	11.3 [8.3]
eGFR, ml/min per 1.73m^2	86 [21]	81 [20]
Year of treatment initiation, 0 = 2013, … 10 = 2023	4.7 [3.1]	4.7 [2.6]
Number of current glucose-lowering drugs	1.7 [0.9]	1.8 [0.9]
Number of ever prescribed glucose-lowering drugs	2.1 [1.0]	2.3 [1.1]
History of ASCVD*		
Previous event	73 (12%)	162 (16%)
No previous event	517 (88%)	840 (84%)
History of Heart Failure		
Yes	3 (0.5%)	15 (1.5%)
No	587 (99.5%)	987 (98.5%)
Systolic blood pressure, mmHg	138 [17]	137 [16]
Diastolic blood pressure, mmHg	82 [10]	80 [9]
Cholesterol, mg/dl	189 [47]	185 [45]
Triglycerides, mg/dl	215 [117]	214 [133]
Smoking status		
Active smoker	37 (6%)	69 (7%)
Not an active smoker	553 (94%)	933 (93%)
Lipid lowering drug prescription		
Yes	146 (25%)	343 (34%)
No	444 (75%)	659 (66%)
Blood pressure lowering drug prescription		
Yes	269 (46%)	539 (54%)
No	321 (54%)	463 (46%)
Insulin treatment		
Yes	273 (46%)	429 (43%)
No	317 (54%)	573 (57%)

Data are mean [SD] and number (%)
*Myocardial infarction, unstable angina, stable angina, coronary revascularization, stroke, transient ischemic attack or peripheral artery disease
ASCVD: atherosclerotic cardiovascular disease; BMI: body mass index; DPV: Diabetes Prospective Follow-up; eGFR: estimated Glomerular Filtration Rate; GLP-1-RA: GLP-1 receptor agonists; HbA1c: hemoglobin A1c; SGLT2i: SGLT2-inhibitors.

sTable 6 Baseline clinical characteristics of individuals who were recommended GLP-1 receptor agonists (GLP-1-RA) or SGLT2-inhibitors (SGLT2i) treatment based on the parsimonious personalized treatment selection model in the 40% model validation sample from the Diabetes Prospective Follow-up (DPV) registry.

	**GLP-1-RA-optimal individuals (n=851)**	**SGLT2i-optimal individuals (n=741)**
Age, years	65 [11]	57 [12]
Sex		
Male	460 (54%)	475 (64%)
Female	391 (46%)	266 (36%)
BMI, kg/m^2	35.7 [7.1]	32 [5.6]
HbA1c, %	7.8 [1.4]	7.8 [1.6]
Diabetes duration, years	12.3 [8.8]	10.0 [7.4]
eGFR, ml/min per 1.73m^2	70 [17]	97 [13]
Year of treatment initiation, 0 = 2013, … 10 = 2023	4.8 [2.8]	4.6 [2.8]
Number of current glucose-lowering drugs	1.8 [0.9]	1.8 [0.9]
Number of ever prescribed glucose-lowering drugs	2.2 [1.1]	2.2 [1.2]
History of ASCVD*		
Previous event	75 (9%)	160 (22%)
No previous event	776 (91%)	581 (78%)
History of Heart Failure		
Yes	17 (1.9%)	1 (0.1%)
No	834 (98.1%)	740 (99.9%)
Systolic blood pressure, mmHg	138 [17]	137 [16]
Diastolic blood pressure, mmHg	80 [10]	82 [9]
Cholesterol, mg/dl	183 [44]	191 [48]
Triglycerides, mg/dl	218 [123]	210 [132]
Smoking status		
Active smoker	33 (4%)	73 (10%)
Not an active smoker	818 (96%)	668 (90%)
Lipid lowering drug prescription		
Yes	263 (31%)	226 (30%)
No	588 (69%)	515 (70%)
Blood pressure lowering drug prescription		
Yes	463 (54%)	345 (47%)
No	388 (46%)	396 (53%)
Insulin treatment		
Yes	397 (46%)	305 (43%)
No	454 (54%)	436 (57%)

Data are mean [SD] and number (%)
*Myocardial infarction, unstable angina, stable angina, coronary revascularization, stroke, transient ischemic attack or peripheral artery disease
ASCVD: atherosclerotic cardiovascular disease; BMI: body mass index; DPV: Diabetes Prospective Follow-up; eGFR: estimated Glomerular Filtration Rate; GLP-1-RA: GLP-1 receptor agonists; HbA1c: hemoglobin A1c; SGLT2i: SGLT2-inhibitors.

sTable 7 Internal model validation results (concordant-discordant analysis) of the full model that included all tailoring variables. Estimates incl. 95% CIs from the exponential Accelerated Failure Time (AFT) validation model are shown.

	**Concordant**	**Discordant**
**Overall**	n = 816	n = 776
Time factor (AFT)	1.09 [0.89; 1.40]	-
Hazard Ratio	0.92 [0.67; 1.26]	-
**GLP-1-RA-optimal**	n = 329	n = 515
Time factor (AFT)	1.23 [0.76; 1.98]	-
Hazard Ratio	0.81 [0.50; 1.31]	-
**SGLT2i-optimal**	n = 487	n = 261
Time factor (AFT)	1.14 [0.69; 1.89]	-
Hazard Ratio	0.88 [0.53; 1.45]	-

AFT: Accelerated Failure Time.
